# The Jun/miR-22/HuR regulatory axis contributes to tumourigenesis in colorectal cancer

**DOI:** 10.1186/s12943-017-0751-3

**Published:** 2018-01-19

**Authors:** Yanqing Liu, Xiaorui Chen, Rongjie Cheng, Fei Yang, Mengchao Yu, Chen Wang, Shufang Cui, Yeting Hong, Hongwei Liang, Minghui Liu, Chihao Zhao, Meng Ding, Wu Sun, Zhijian Liu, Feng Sun, Chenyu Zhang, Zhen Zhou, Xiaohong Jiang, Xi Chen

**Affiliations:** 1 State Key Laboratory of Pharmaceutical Biotechnology, Collaborative Innovation Center of Chemistry for Life Sciences, Jiangsu Engineering Research Center for MicroRNA Biology and Biotechnology, NJU Advanced Institute for Life Sciences (NAILS), School of Life Sciences, Nanjing University, Nanjing, Jiangsu 210046 China; 2 Tianjin Medical University Cancer Institute and Hospital, National Clinical Research Center of Cancer, Key Laboratory of Cancer Prevention and Therapy, Tianjin, 300060 China; 3 Department of Gastrointestinal Surgery, Nanjing Drum Tower Hospital, The Affiliated Hospital of Nanjing University Medical School, Nanjing, Jiangsu 210008 China

**Keywords:** Colorectal cancer, HuR, miR-22, Jun, Proliferation, Migration

## Abstract

**Background:**

Colorectal cancer (CRC) is a severe health problem worldwide. Clarifying the mechanisms for the deregulation of oncogenes and tumour suppressors in CRC is vital for its diagnosis, treatment, prognosis and prevention. Hu antigen R (HuR), which is highly upregulated in CRC, functions as a pivotal oncogene to promote CRC progression. However, the underlying cause of its dysregulation is poorly understood.

**Methods:**

In CRC tissue sample pairs, HuR protein levels were measured by Western blot and immunohistochemical (IHC) staining, respectively. HuR mRNA levels were also monitored by qRT-PCR. Combining meta-analysis and microRNA (miRNA) target prediction software, we predicted miRNAs that targeted HuR. Pull-down assay, Western blot and luciferase assay were utilized to demonstrate the direct binding of miR-22 on HuR’s 3’-UTR. The biological effects of HuR and miR-22 were investigated both in vitro by CCK-8, EdU and Transwell assays and in vivo by a xenograft mice model. JASPAR and SABiosciences were used to predict transcriptional factors that could affect miR-22. Luciferase assay was used to explore the validity of putative Jun binding sites for miR-22 regulation. ChIP assay was performed to test the Jun’s occupancy on the C17orf91 promoter.

**Results:**

We observed a significant upregulation of HuR in CRC tissue pairs and confirmed the oncogenic function of HuR both in vitro and in vivo. We found that an important tumour-suppressive miRNA, miR-22, was significantly downregulated in CRC tissues and inversely correlated with HuR in both CRC tissues and CRC cell lines. We demonstrated that miR-22 directly bound to the 3’-UTR of HuR and led to inhibition of HuR protein, which repressed CRC proliferation and migration in vitro and decelerated CRC xenografted tumour growth in vivo. Furthermore, we found that the onco-transcription factor Jun could inhibit the transcription of miR-22.

**Conclusions:**

Our findings highlight the critical roles of the Jun/miR-22/HuR regulatory axis in CRC progression and may provide attractive potential targets for CRC prevention and treatment.

**Electronic supplementary material:**

The online version of this article (10.1186/s12943-017-0751-3) contains supplementary material, which is available to authorized users.

## Background

Colorectal cancer (CRC) is one of the most malignant cancer types around the world due to its high morbidity and mortality [1, 2]. Many risk factors for CRC have been identified, including smoking, obesity, unhealthy diet, *Helicobacter pylori* infection, physical inactivity and precancerous lesions [3, 4]. Among these various causes of CRC, the aberrant activation or upregulation of oncogenes (e.g., KRAS [5] or EGFR [6]) and loss of function or downregulation of tumour suppressors (e.g., PDCD4 [7] or TIA1 [8]) are central. A better understanding of the underlying mechanism for the abnormal development of CRC is vital to the diagnosis, treatment, prognosis and prevention of this disease.

RNA binding proteins (RBPs) are heterogeneous sets of proteins with essential roles in RNA metabolism and post-transcriptional gene regulation [9]. Scientists have identified more than 500 RBPs, some of which are tightly linked to the initiation and progression of human cancers [9, 10]. Hu antigen R (HuR), also known as embryonic lethal abnormal vision 1 (ELAVL1), is one of the most famous cancer-related RBPs [11–13]. HuR mainly localises to the nucleus, until the cell receives one of various stimulations that promote the translocation of HuR to the cytoplasm [14], where HuR uses three RRMs (RNA recognition motifs) to bind UTRs (untranslated regions) of downstream mRNAs at their AREs (AU-rich elements). Through this mechanism of interaction, HuR stabilizes target mRNAs or promotes their translation, yet HuR occasionally represses the translation of some targets [12, 14]. A considerable number of target mRNAs of HuR encode proteins essential for cell survival and proliferation (e.g., CCNA [15], HIF1A [16], COX-2 [17] and VEGF [18]), and therefore HuR plays an oncogenic role in the development and progression of various cancers [11–14]. For example, HuR promotes tumour cell growth by stabilizing Bcl-2 in glioblastoma [19]. In breast cancer, the binding of HuR to CCNE1’ 3’-UTR significantly increases the mRNA stability and protein half-life of CCNE1, thus promoting breast cancer proliferation [20]. In addition, HuR affects the metastasis of oral cancer cells [21]. For CRC, the roles of HuR have also been intensively investigated. HuR was reported to be upregulated in CRC [22–24] and stabilizes many oncogenes (e.g., COX-2 [24], VEGF [25] and IL-8 [25]), leading to enhanced CRC cell growth and tumourigenicity. Another study found a robust correlation between increased cytoplasmic HuR levels with COX-2 expression and colon cancer stage [26]. In a nude mouse model of CRC, HuR significantly promotes xenografted tumour growth [22]. In summary, these studies support the oncogene role of HuR in CRC. However, the underlying mechanism for the aberrant expression of HuR in CRC is poorly understood.

Among various regulatory mechanisms for gene expression, microRNAs (miRNAs) are highlighted as a prominent and intriguing one due to their extensive expression and functions in widespread organisms and biological activities [27], including tumourigenesis [28]. During tumourigenesis, many miRNAs undergo changes in expression, thus negatively regulating their cancer-related target genes to affect tumour phenotypes. These miRNAs are referred to as oncomiRs or tumour-suppressive miRNAs [28]. miR-22 is known as one of the most important tumour-suppressive miRNAs in many different cancer types [29, 30]. In hepatocellular carcinoma, miR-22 suppresses cell proliferation and tumourigenicity and is correlated with patient prognosis [31]. In breast cancer, miR-22 inhibits cell invasion and migration by targeting Sp1, CD147 and GLUT1 [32, 33]. In gastric cancer, miR-22 inhibits both tumour proliferation and metastasis by targeting MMP14 and Snail [34]. For CRC, miR-22 even has a more profound tumour-suppressive effect. miR-22 is significantly downregulated in CRC tissue compared with that in normal adjacent mucosa [35] and improves 5-FU and paclitaxel sensitivity in chemotherapy [36, 37]. Overexpression of miR-22 inhibits HIF-1α and VEGF, thus suppressing CRC cell angiogenesis [38]. miR-22 is also activated by vitamin D and exerts anti-proliferative and anti-migratory roles in CRC cells by targeting TIAM1, MMP-2 and MMP-9 [39, 40]. Although miR-22 shows vital significance in CRC, the exact mechanism through which miR-22 influences CRC progression is far from understood.

In this study, we showed that upregulated HuR functions as a potent oncogene in promoting CRC proliferation and migration and is a target gene of miR-22. We also found that miR-22 inhibits CRC cell proliferation and migration in vitro and decelerates xenografted tumour growth in vivo by targeting HuR. Moreover, the onco-transcription factor Jun was found to suppress miR-22 expression at the transcriptional level. Thus, the Jun/miR-22/HuR regulatory axis may contribute to tumourigenesis of colorectal cancer.

## Methods

### Tissue samples

CRC tissues were collected from patients who underwent surgical resection at the Affiliated Drum Tower Hospital of Nanjing University Medical School (Nanjing, China). All patients signed consent letters and all manipulation of the tissues was approved by the Ethics Committee of Nanjing University. After surgery, the tissue samples were immediately frozen in liquid nitrogen and stored at −80 °C. All experiments were performed in accordance with The Code of Ethics of the World Medical Association (Declaration of Helsinki) and the guidelines of the Nanjing University. Clinical features of the patients are listed in Additional file 1: Table S1.

### Cell culture and transfection

All cell lines used in this study were purchased from the Shanghai Institute of Biochemistry and Cell Biology, Chinese Academy of Sciences (Shanghai, China) and verified by short-tandem repeat (STR) profiling. All cells were cultured in the appropriate medium (RPMI-1640 for NCM460, SW480, HT29, HCT15 and HCT116; L-15 for SW620; DMEM for Caco2; and F-12 K for LOVO) supplemented with 10% FBS (Gibco, Carlsbad, CA, USA) in a humidified atmosphere with 5% CO_2_ at 37 °C. Lipofectamine 2000 (Invitrogen, Carlsbad, CA, USA) was used for transient transfection of small RNA oligos and plasmids. For miRNA overexpression or knockdown, miRNA mimics or inhibitors (GenePharma, Shanghai, China) were used, respectively. For protein overexpression or knockdown, gene-specific overexpression vectors (FulenGen, Guangzhou, China) or siRNAs (GenePharma) were used, respectively. The siRNA sequences are listed in Additional file 2: Table S2.

### Online database analysis

Targetscan [41] (http://www.targetscan.org/vert_71/) was used to predict potential miRNAs that could target HuR. Oncomine database (https://www.oncomine.org/resource/login.html) [42] was utilised to analyse the HuR expression level in CRC patients from a TCGA cohort. We adopted a Cancer vs. Normal Analysis to compare the expression levels of HuR in normal colon and rectum with those of colon adenocarcinoma and rectal adenocarcinoma. To explore the association between HuR or miR-22/miR-129 expression levels and the life expectancy of CRC patients, we downloaded RNA-Seq raw data and survival data of CRC patients from the TCGA data portal (http://cancergenome.nih.gov/). We utilised Kaplan-Meier curves to compare overall survival differences between “high” and “low” expression groups and calculated *p* values using the log-rank test in the survival package in R. To predict transcriptional factors that could affect miR-22, JASPAR (http://jaspar.binf.ku.dk/) [43] and SABiosciences [44] (http://www.sabiosciences.com/chipqpcrsearch.php) were used.

### Protein isolation and western blot

Total protein was extracted with RIPA lysis buffer (Beyotime, China) supplemented with the proteinase inhibitors PMSF (Roche, USA) and PI (Thermo, USA). Proteins were separated by 10% SDS-PAGE (Bio-Rad, USA). GAPDH was used as an internal control. Antibodies against HuR and GAPDH were purchased from Santa Cruz Biotechnology (sc-5261 and sc-365,062, respectively) and the antibody against Jun was purchased from CST (#9165).

### RNA isolation and qRT-PCR

TRIzol reagent (Sigma, St. Louis, USA) was used for total RNA extraction. TaqMan miRNA Assay Primers (Applied Biosystems, USA) or oligo d(T)18 primers (TaKaRa, Japan) were used for reverse transcription of miRNAs and protein-coding genes, respectively. To generate fluorescence signal in qRT-PCR, TaqMan miRNA Assay Probes (Applied Biosystems, USA) and SYBR Green dye (Ambion, Carlsbad, CA, USA), combined with gene-specific primer pairs, were used for miRNA and protein-coding gene quantification, respectively. After the qRT-PCR procedure, we set a fixed threshold for the cycle threshold (C_T_) data, and the mean C_T_ was determined from triplicate reaction wells. U6 snRNA or GAPDH was used as an internal control (IC) for miRNAs or protein-coding genes, respectively, and the relative change in the level of target genes (TGs) normalised to IC between experimental groups (EGs) and the control group (CG) was calculated with the eq. 2^-ΔΔCT^, in which ΔΔC_T_ = (C_T TG_− C_T IC_)_EG_ − (C_T TG_ − C_T IC_)_CG_. All sequences of the primers used are listed in Additional file 2: Table S2.

### Pull-down assay

The pull-down assay was carried out according to a previously described protocol [8]. Briefly, a DNA probe complementary to HuR mRNA and labelled with biotin at the 3′ terminal, was synthesised to pull down HuR mRNA. A scrambled biotinylated probe was used as a negative control (Genescript, Nanjing, China). The probes were incubated with streptavidin-coated magnetic beads (New England BioLabs, USA) and then with SW480 lysate. After incubation, beads were washed and treated with Trizol reagent to extract RNA. The sequence of the probe is listed in Additional file 2: Table S2.

### Luciferase assay

Luciferase vectors used in this study were purchased from Genescript (China). Briefly, for miRNA binding site tests, pMIR-report luciferase vectors containing binding sites for miR-22 or miR-129 on HuR’s 3’-UTR were constructed. We also purchased mutant plasmid to test binding specificity. The miR-22 binding site was mutated from GGCAGCT to CCGTCGA, and the binding site of miR-129 was mutated from CAAAAA to GTTTTT. For the miR-22 promoter assay, miR-22 promoter regions containing different Jun binding sites were inserted into pGL3 basic reporter vectors (Promega, USA). When transfecting SW480 with luciferase vectors and small RNA oligos, we also co-transfected the cells with a β-galactosidase (β-gal) expression vector (Ambion) as a control. Luciferase activity was tested using a luciferase assay kit (Promega, USA).

### Cell proliferation assay

To measure the proliferation rate of SW480, we conducted CCK-8 and EdU assays according to protocols described elsewhere [8]. Briefly, SW480 was seeded in 6-well plates and transfected with small RNA oligos or plasmids. At 24 h after transfection, cells were harvested and reseeded in 96-well plates for CCK-8 or 48-well plates for EdU assays, respectively. For the CCK-8 assay, Cell Counting Kit-8 (Dojindo, Japan) was added into cells at the following time points: 12, 24, 36, 48 and 60 h after reseeding. After incubation for 2 h, the absorbance was measured at a wavelength of 450 nm. For the EdU assays, an EdU assay kit (RiBoBio, China) was used to determine the proliferation rate of cells according to the manufacturer’s instructions.

### Cell migration assay

SW480 was transfected with small RNA oligos or plasmids. After 24 h, cells were resuspended in FBS-free RPMI-1640 medium and reseeded on the upper surface of 24-well Millicell plates (Millipore, USA). Cells were allowed to migrate across the 8-μm membrane toward medium with 20% FBS for 24 h. Then, the cells were fixed with 4% paraformaldehyde and dyed with 0.5% crystal violet. Nonmigrating cells were removed using a cotton swab. The migrant cells were blindly counted under a light microscope (BX51 Olympus, Japan).

### Chromatin immunoprecipitation (ChIP) assay

The ChIP assay was performed using a commercial kit (Beyotime, Shanghai, China) according to the manufacturer’s instructions. An antibody against Jun was used to immunoprecipitate Jun-chromatin complexes. Anti-IgG (Santa Cruz, USA) served as a negative control. The ChIP products were amplified by PCR and then separated on 1.5% agarose gels. The primers for amplification are listed in Additional file 2: Table S2.

### Mouse experiments

To explore tumour growth in vivo, SW480 overexpressing or knocked-down for the corresponding small RNA or protein and control cells were injected into nude mice (purchased from the Model Animal Research Center of Nanjing University) in their armpits. Mice were sacrificed after 25 days or 30 days, and tumours were removed for RNA and protein extraction, haematoxylin and eosin (H&E) staining or immunohistochemical (IHC) staining. All animal experiments complied with the ARRIVE guidelines and were carried out in accordance with the National Institutes of Health guide for the care and use of Laboratory animals (NIH Publications No. 8023, revised 1978) and the guidelines of the Nanjing University.

### Statistical analysis

All experiments were performed at least in triplicate, and each individual experiment was repeated several times. Student’s *t*-test was used to analyse differences between two groups. *P* values less than 0.05 were considered statistically significant.

## Results

### HuR is significantly upregulated in CRC tissues and functions as an oncogene in CRC

**Fig. 1:**
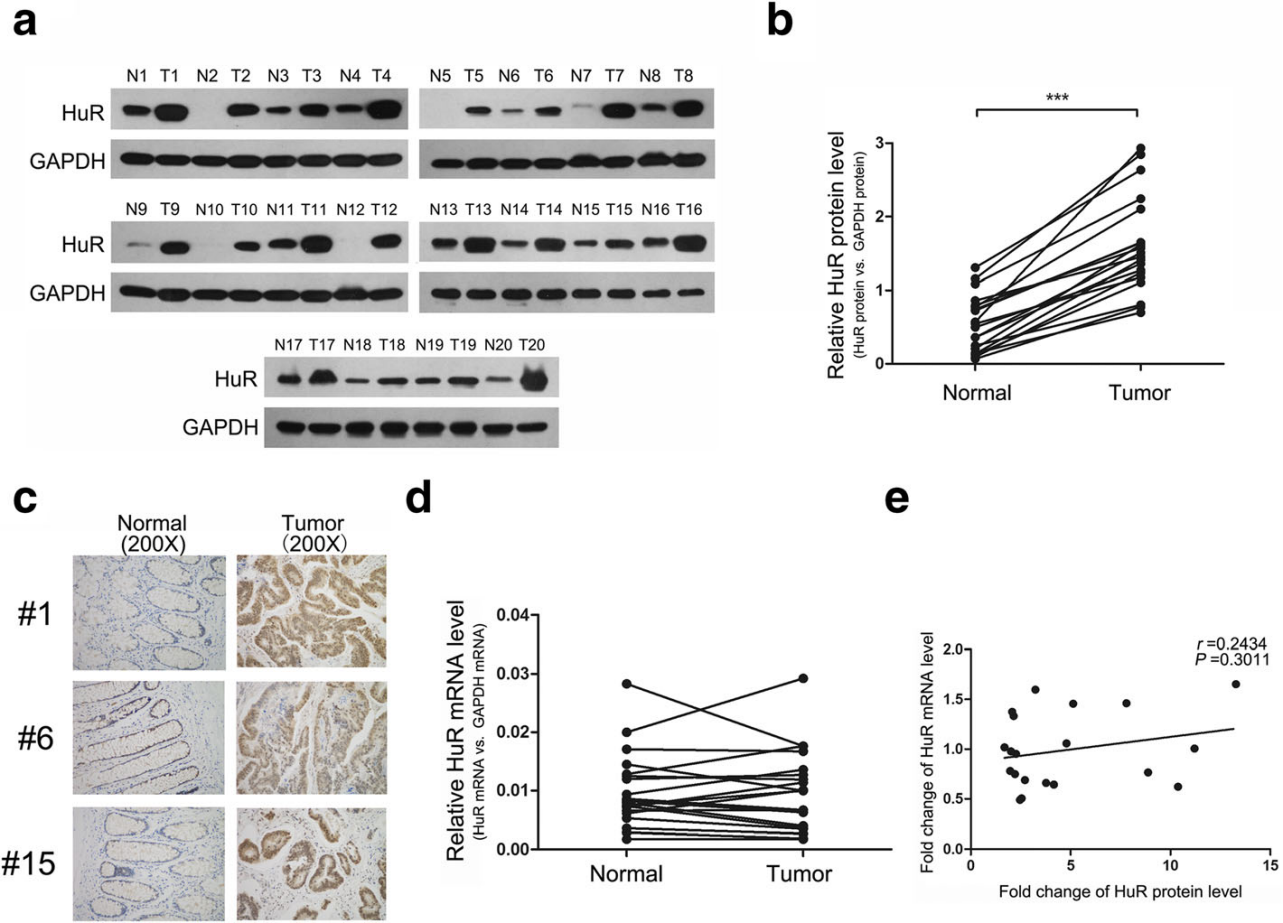
HuR protein but not mRNA is significantly upregulated in CRC tissues. **a** and **b** Western blot analysis of HuR levels in CRC tissue pairs. **c** IHC staining of HuR in CRC tissue pairs. **d** qRT-PCR analysis of HuR mRNA levels in CRC tissue pairs. **e** Pearson’s correlation scatter plot of the fold changes of HuR protein and mRNA levels in CRC tissue pairs. ***P < 0.001

First, the expression levels of HuR in CRC tissues and adjacent normal tissues were examined. The online database Oncomine [42] was utilised to analyse HuR expression in CRC patients from the TCGA dataset. As shown in Additional file 3: Figure S1a, HuR in both colon and rectal adenocarcinoma showed increased expression levels compared with that in normal colon or rectum. This result was validated using 20 paired CRC tissues, in which HuR protein levels were found to be significantly upregulated in cancer tissues compared with those in normal tissues (Fig. 1a and b). Moreover, we randomly selected 3 paired CRC tissues to perform IHC staining for HuR. HuR expression is low in normal tissues, but significantly elevated in cancerous tissues (Fig. 1c). In contrast, HuR mRNA levels showed irregular alteration between cancer tissues and adjacent normal tissues (Fig. 1d). Pearson’s correlation analysis of scatter plots further revealed an inconsistent relationship between HuR protein levels and mRNA levels in these tissues (Fig. 1e). Additionally, we analysed the correlation between HuR expression levels and survival times of patients in the TCGA database. High HuR expression was found to be significantly correlated with poor survival of CRC patients (Additional file 3: Figure S1b). Taken together, these results suggested an oncogenic role of HuR in CRC.

To investigate whether HuR is involved in CRC proliferation and migration, HuR-specific siRNA (si-HuR) was used to silence HuR expression in the CRC cell line SW480. The efficiency of si-HuR is shown in Additional file 4: Figure S2a. In the CCK-8 assay, interference of HuR expression greatly reduced the proliferation rate of SW480 cells (Additional file 5: Figure S3a). Analogous results were observed in an EdU assay (Additional file 5: Figure S3b and c). We next performed a Transwell assay to investigate the influence of HuR on SW480 cell migration. As plotted in Additional file 5: Figure S3d and e, the number of migrant cells in the HuR silencing group was markedly less than the control group, indicating that HuR promoted SW480 migration.

To explore the effect of HuR on CRC in vivo, we infected SW480 with si-HuR lentivirus to stably knockdown HuR in SW480, and then injected the infected cells into nude mice to establish a CRC xenograft mouse model. Consistent with in vitro results, HuR silencing (Additional file 5: Figure S3i) significantly decelerated xenografted tumour growth (Additional file 5: Figure S3f-h). We also sent the tumours for H&E and IHC staining (Additional file 5: Figure S3j and k). IHC staining for HuR and Ki-67 showed less HuR protein and reduced proliferative activity, while H&E staining revealed reduced mitosis in the HuR knockdown group compared with that in the control group. These results demonstrated that HuR promoted CRC proliferation and migration in vitro and accelerated tumour growth in vivo.

### HuR is a potential target gene of miR-22 and miR-129

**Fig. 2:**
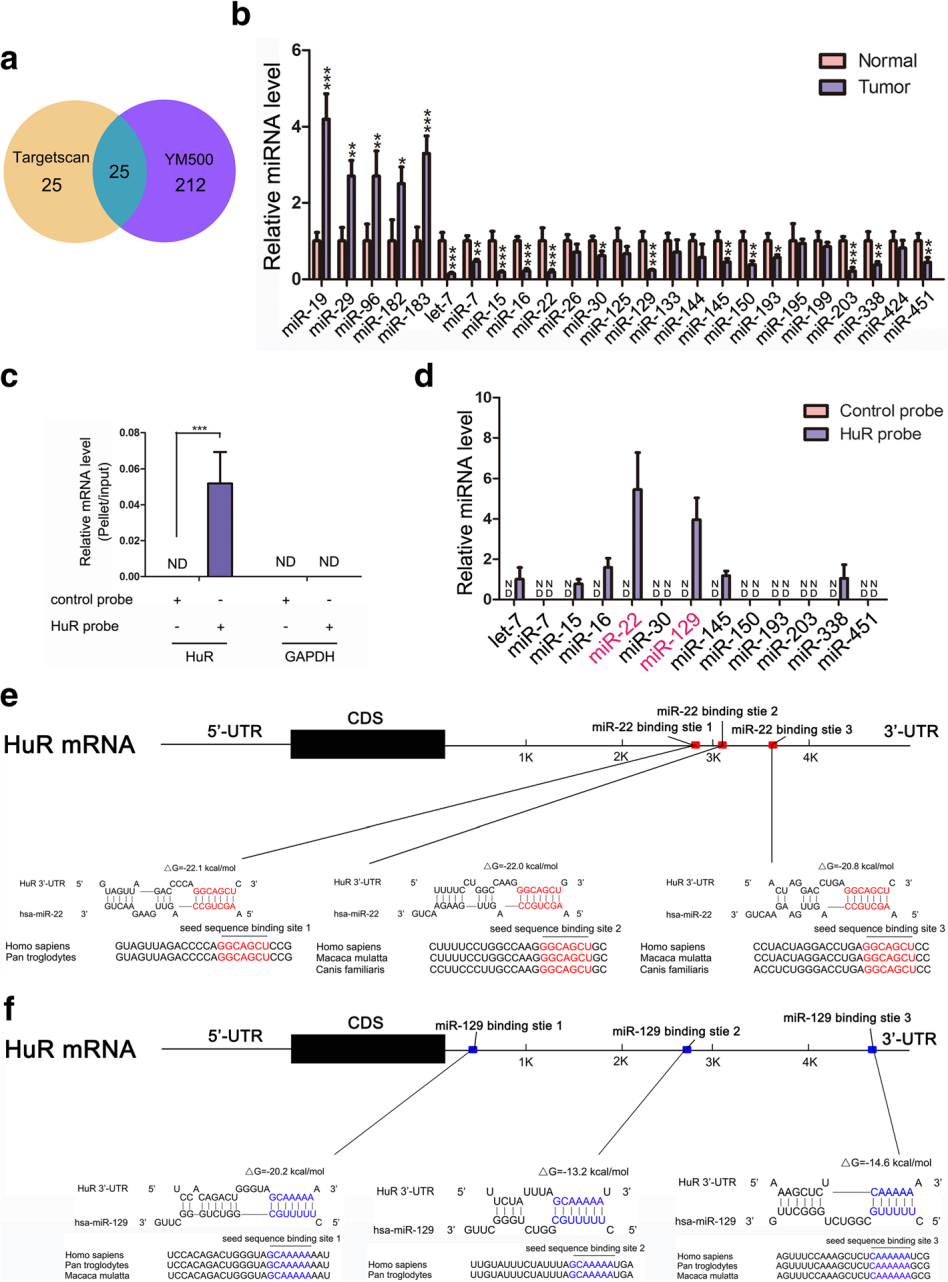
HuR is a potential target gene of miR-22 and miR-129. **a** A Venn diagram was used to search for potential miRNAs that could target HuR in CRC. **b** Levels of candidate miRNAs in CRC tissue pairs. **c** Efficiency and specificity of the HuR probe. **d** Pull-down assays showed that among the downregulated 13 candidate miRNAs, 7 could bind to HuR mRNA. **e** and **f** Schematic descriptions of the hypothetical duplexes formed by miR-22 or miR-129 with the 3’-UTR of HuR. *P < 0.05; **P < 0.01; ***P < 0.001

The inconsistency between HuR protein and mRNA levels (Fig. 1e) implied that some post-transcriptional gene regulation mechanisms were involved in the control of HuR expression. Since miRNAs represent a common post-transcriptional mechanism for gene regulation in different physiological and pathological circumstances, we hypothesised that some miRNAs might target HuR in CRC. First, the bioinformatics software TargetScan [41] was used to predict miRNAs that might target HuR. As shown in Fig. 2a, 50 miRNAs were identified as possible candidates. Previously, we identified 237 miRNAs that were significantly altered in CRC samples using computational algorithm YM500 [8] (Fig. 2a). By sorting the overlap between Targetscan results and YM500 results, 25 miRNAs common to both sets were chosen for further verification (Fig. 2a). A qRT-PCR assay was performed to validate the expression levels of the 25 selected miRNAs in CRC tissues compared with those in adjacent normal tissues. As shown in Fig. 2b, 5 miRNAs were significantly upregulated, 13 were decreased, and the other 7 were not different between CRC tissue pairs. Given that miRNA levels should have an opposite trend compared to their target genes, we focused on the 13 downregulated miRNAs.

To examine if the 13 candidates could bind directly with HuR mRNA, a biotinylated HuR probe was used to pull down HuR mRNA (Fig. 2c) and the 13 selected miRNAs were measured in the HuR mRNA-containing pellet using qRT-PCR. As shown in Fig. 2d, 7 of the 13 miRNAs were detected in the pellet, indicating that they could bind directly with HuR mRNA and might suppress HuR protein expression. Among them, miR-22 and miR-129 showed the highest enrichments. Therefore, we chose miR-22 and miR-129 for further investigation.

As predicted by Targetscan, miR-22 and miR-129 have 3 conserved binding sites in the 3’-UTR of HuR (Fig. 2e and f). The minimum free energy values of the miR-22-HuR mRNA hybridisations were −22.1, −22.0 and −20.8 kcal/mol, which were lower than that of miR-129-HuR mRNA duplexes (−20.2, −13.2 and −14.6 kcal/mol), indicating that miR-22 has a tighter interaction with HuR mRNA than miR-129 (Fig. 2e and f).

### miR-22 and miR-129 can inhibit HuR by binding to its 3’-UTR

**Fig. 3:**
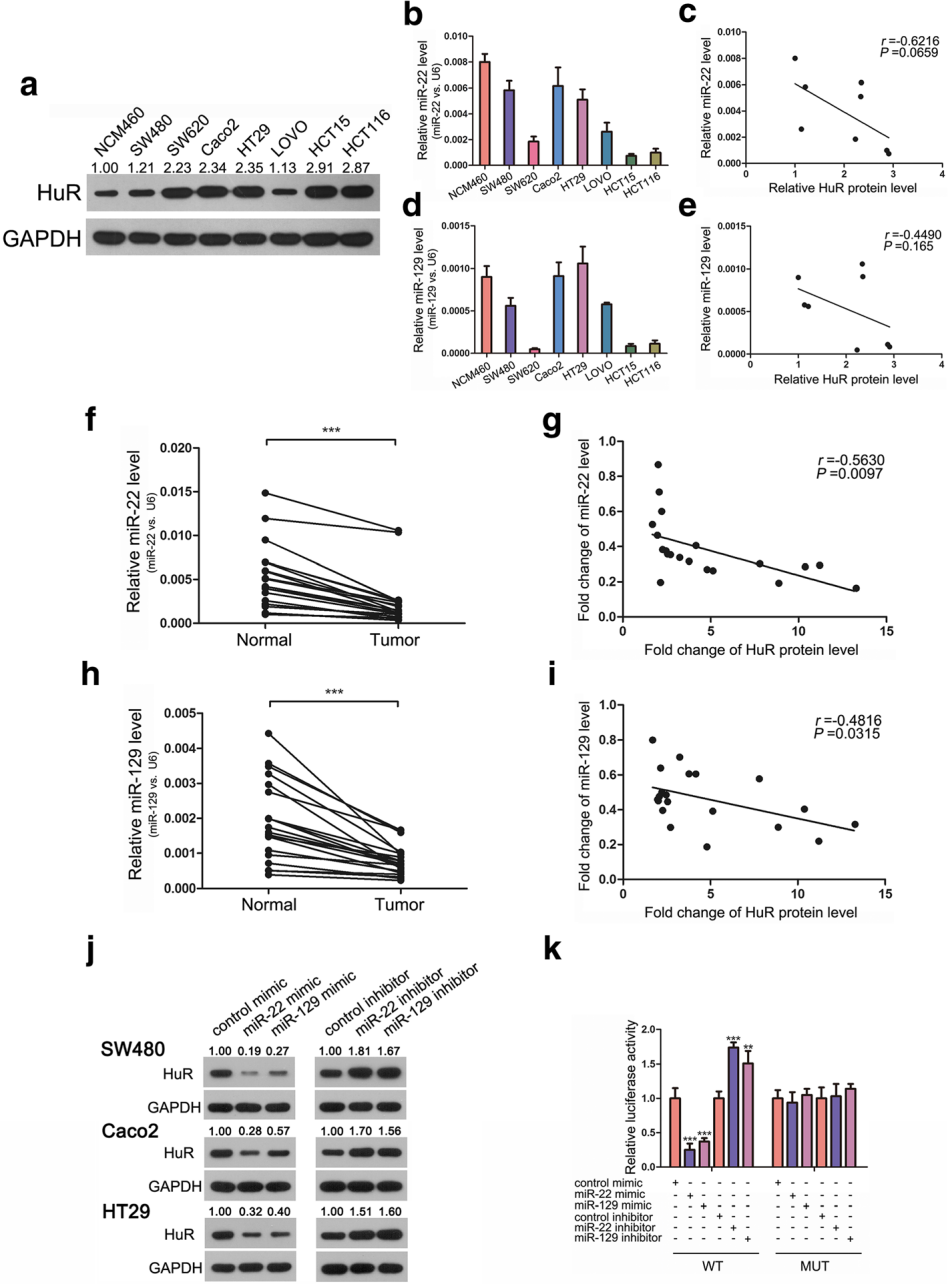
miR-22 and miR-129 can inhibit HuR by binding to its 3’-UTR. **a** Western blot analysis of HuR levels in normal colon mucosal epithelial cell line NCM460 and 7 CRC cell lines. **b**-**e** qRT-PCR analysis of miR-22 and miR-129 levels and the correlation between miRNA and HuR levels in the aforementioned 8 cell lines. **f**-**i** qRT-PCR analysis of miR-22 and miR-129 levels and the correlation between fold changes of miRNA and HuR levels in CRC tissue pairs. **j** Western blot analysis of HuR levels in 3 CRC cell lines after treatment with miR-22/miR-129 mimic or inhibitor. **k** Relative luciferase activities in SW480 treated with a miR-22/miR-129 mimic or inhibitor. **P < 0.01; ***P < 0.001

Subsequently, we measured HuR, miR-22 and miR-129 levels in normal colon mucosal epithelial cell line NCM460 and 7 CRC cell lines (SW480, SW620, Caco2, HT29, LOVO, HCT15 and HCT116). HuR showed higher expression levels in CRC cell lines than those in NCM460, whereas miR-22 and miR-129 expression levels were lower in the CRC cell lines (Fig. 3a, b and d). Further analysis using Pearson’s correlation analysis of scatter plots revealed that miR-22 and miR-129 were inversely correlated with HuR expression (Fig. 3c and e). We also analysed the correlation between the levels of miR-22/miR-129 and HuR in the CRC tissues mentioned above. As shown in Fig. 3f–i, miR-22 and miR-129 were inversely associated with the level of HuR in CRC tissues. Kaplan-Meier curves showed that higher miR-22 or miR-129 levels predicted longer survival in CRC patients, which was contrary to that of HuR (Additional file 6: Figure S4a and b).

To determine whether miR-22 or miR-129 could inhibit HuR expression, SW480 cells were transfected with mimics or inhibitors of these two miRNAs to alter their cellular levels (Additional file 6: Figure S4c and d). As anticipated, mimics of miR-22 and miR-129 both inhibited HuR expression, while inhibitors of them increased HuR levels (Fig. 3j). We repeated this experiment in two other CRC cell lines, Caco2 and HT29, and observed similar results (Fig. 3j).

Finally, luciferase assays were conducted to determine if the predicted seed sequence binding sites caused the miRNA-mRNA interaction. DNA fragments containing the miR-22 or miR-129 binding sites of HuR 3’-UTR were inserted into the pMIR-Report Luciferase vector. Then, we co-transfected SW480 cells with this vector, β-gal vector and miR-22/miR-129 mimics/inhibitors. As shown in Fig. 3k, ectopic expression of these two miRNAs dramatically decreased luciferase activity, whereas inhibition of them increased the fluorescence intensity. We then constructed two mutant luciferase vectors on which the binding sites of miR-22/miR-129 in the HuR 3’-UTR were mutated to abolish the interaction between miR-22/miR-129 and HuR mRNA. We used the mutant plasmids to repeat the luciferase experiments, and miR-22 and miR-129 mimics or inhibitors no longer influenced luciferase activity (Fig. 3k). These results indicated that miR-22 and miR-129 can inhibit HuR by binding to its 3’-UTR.

### miR-22 inhibits SW480 proliferation and migration in vitro by targeting HuR

**Fig. 4:**
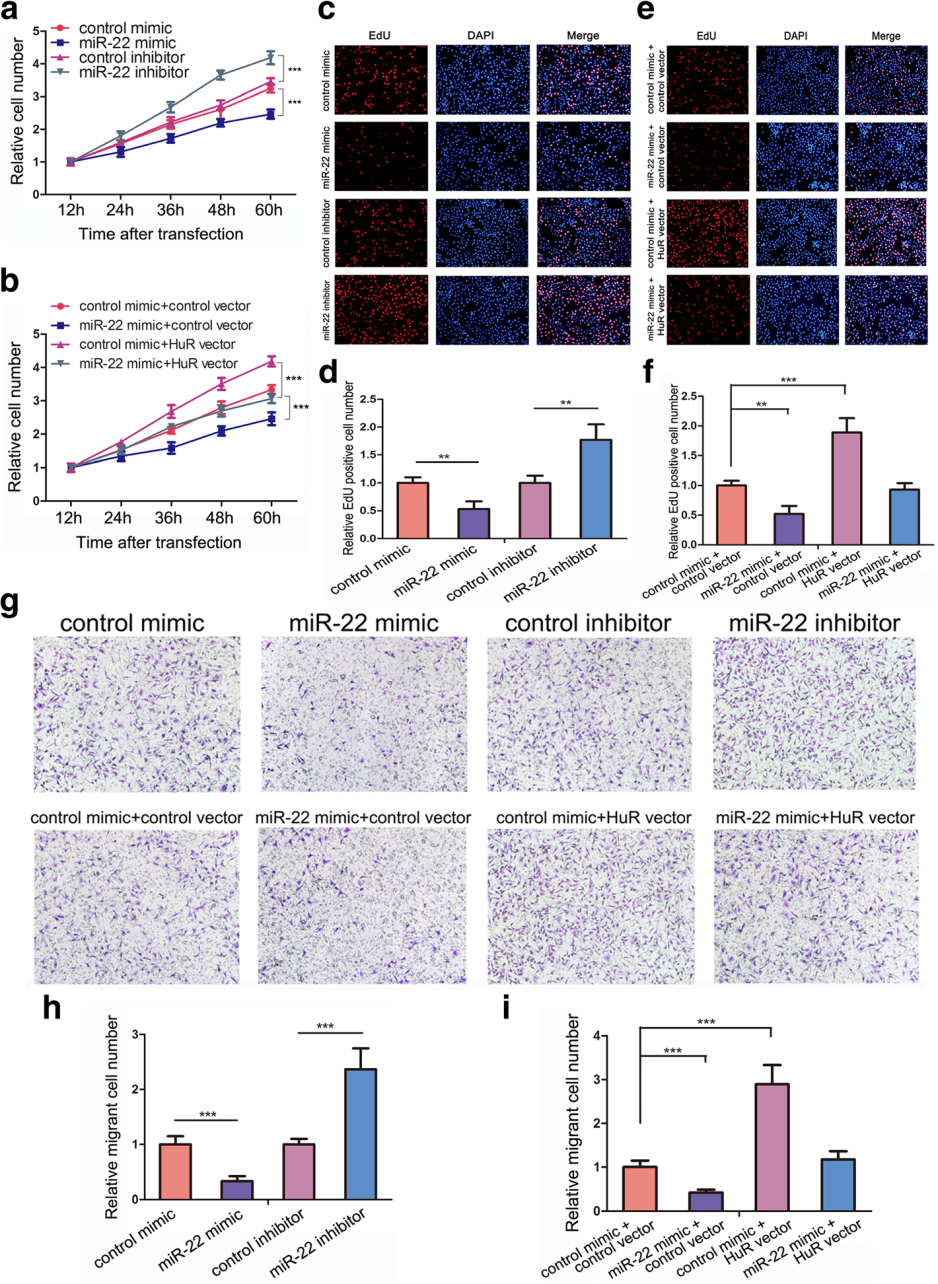
miR-22 inhibits SW480 proliferation and migration in vitro by targeting HuR. **a**, **c** and **d** miR-22 inhibits SW480 proliferation. **a**: CCK-8 assay; **c** and **d**: EdU assay. **b**, **e** and **f** Recovery experiments indicated that the suppression of SW480 proliferation by miR-22 was due to its inhibitory effect on HuR. **b**: CCK-8 assay; **e** and **f**: EdU assay. **g** and **h** Transwell assays revealed that miR-22 could inhibit SW480 migration. **g** and **i** Recovery experiments indicated that the suppression of SW480 migration by miR-22 was due to its inhibitory effect on HuR. **P < 0.01; ***P < 0.001

Considering that miR-22 had a greater inhibitory effect on HuR than miR-129, we next focused on miR-22 to explore the consequences of miR-22-driven HuR suppression in CRC. To test the effect of miR-22 on SW480 cell proliferation, a miR-22 mimic or inhibitor was transfected into SW480 cells. CCK-8 and EdU assays revealed that the miR-22 mimic delayed SW480 proliferation, whereas the miR-22 inhibitor accelerated cell proliferation (Fig. 4a, c and d). We also performed Transwell assays and found that overexpression of miR-22 inhibited SW480 cell migration, whereas inhibition of miR-22 promoted migration (Fig. 4g and h).

To examine if the suppression of proliferation and migration of SW480 by miR-22 was due to the targeting of HuR by miR-22, we performed recovery experiments in which a HuR overexpression vector was used to specifically restore HuR expression suppressed by miR-22. The efficiency of the HuR vector is shown in Additional file 4: Figure S2a. As shown in Fig. 4b, restoration of HuR in SW480 completely abolished the proliferation inhibition effect of miR-22. In EdU assays, the HuR vector evidently enhanced the proliferation of SW480 cells that had been repressed by miR-22 (Fig. 4e and f). Significantly, restoration of HuR expression resulted in a higher percentage of migrant cells compared with that in the miR-22 mimic group in Transwell assays (Fig. 4g and i). Taken together, these results confirmed that miR-22 functioned as a tumour-suppressive miRNA to inhibit SW480 proliferation and migration by targeting HuR.

### miR-22 suppresses CRC tumour growth in vivo by targeting HuR

**Fig. 5:**
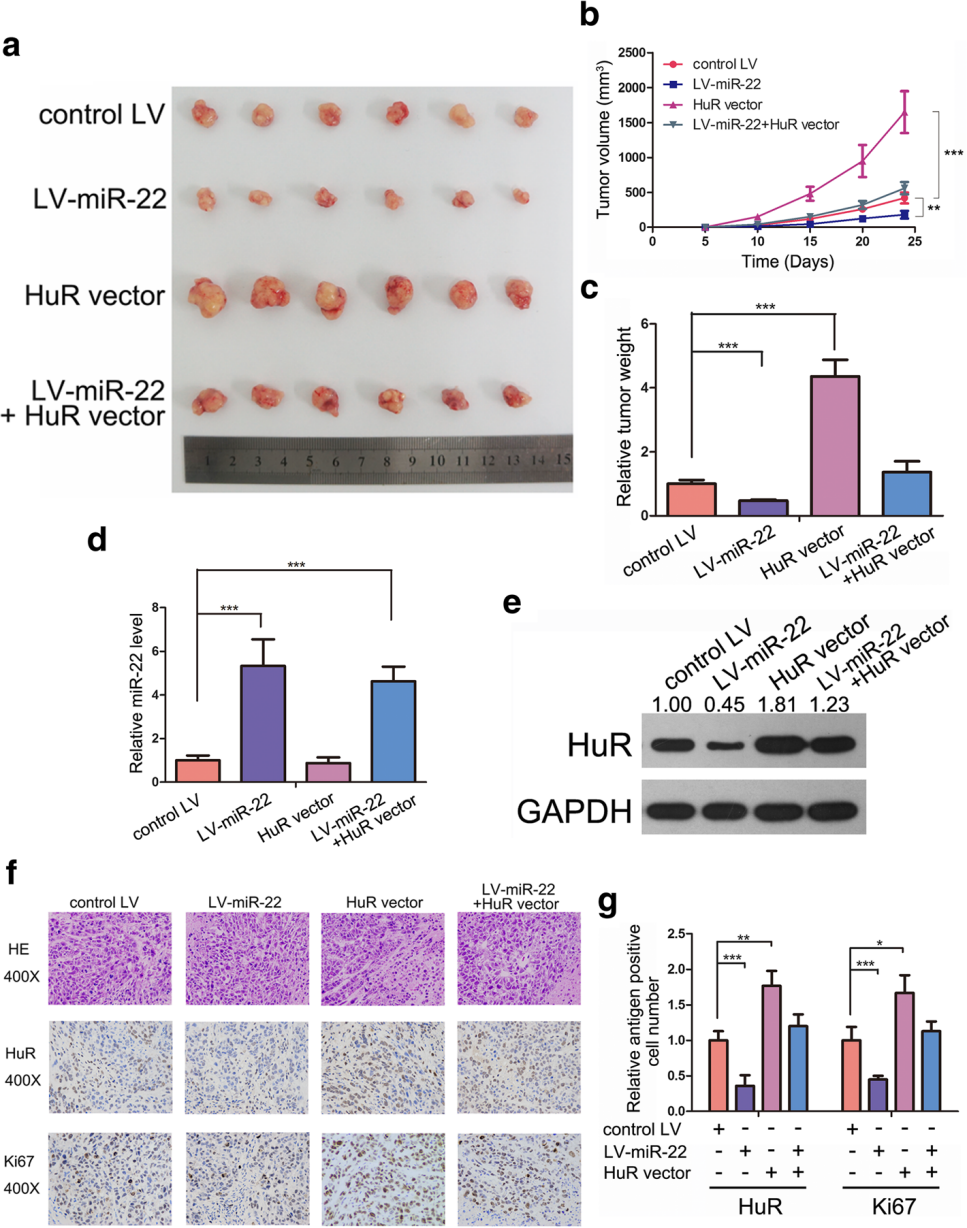
miR-22 suppresses CRC tumour growth in vivo by targeting HuR. **a**-**c** miR-22 slowed down CRC xenografted tumour growth. **a**: Photos of CRC tumours; **b**: Tumour volume curves; **c**: Tumour weights. **d** qRT-PCR analysis of miR-22 levels in CRC xenografted tumours. **e** Western blot analysis of HuR levels in CRC xenografted tumours. **f** and **g** HE staining and IHC staining for HuR and Ki-67 in xenografted tumours. *P < 0.05; **P < 0.01; ***P < 0.001

To validate the contribution of miR-22-induced HuR inhibition on CRC tumourigenesis in vivo, we injected SW480 cells overexpressing miR-22 and/or HuR into the armpits of nude mice to construct a xenograft model for CRC. The growth curve of xenografted tumours showed that overexpression of miR-22 delayed tumour growth, whereas HuR markedly promoted it. Restoration of HuR reversed the inhibition of tumour growth by miR-22 (Fig. 5b). After the mice were sacrificed, the tumours were removed and weighed. As shown in Fig. 5a and c, miR-22 overexpression attenuated xenografted tumour growth, whereas HuR significantly promoted this process. Restoration of HuR diminished the tumour-suppressive effect of miR-22. Total RNA and protein from the tumours were extracted and analysed. As expected, LV-miR-22-infected or HuR vector-transfected groups showed higher miR-22 or HuR levels, respectively, than the control groups (Fig. 5d and e). The HuR vector effectively restored the HuR protein level suppressed by miR-22 (Fig. 5e). H&E staining of these tumours showed decreased cell mitosis in the miR-22-overexpressing group and increased mitosis in the HuR-overexpressing group, whereas xenografts with both miR-22 and HuR overexpression exhibited more cell mitosis than xenografts with miR-22 overexpression alone (Fig. 5f and g). IHC staining for HuR and Ki-67 showed less HuR and lower percentage of proliferative cells in LV-miR-22 infected tumours, whereas tumours overexpressing HuR showed more proliferative cells than the control group. Restoring HuR increased the proliferation rate repressed by LV-miR-22 (Fig. 5f and g). These results revealed the tumour-suppressive role of miR-22 in vivo functioning by targeting HuR.

### miR-22 is inhibited by Jun at the transcription level

**Fig. 6:**
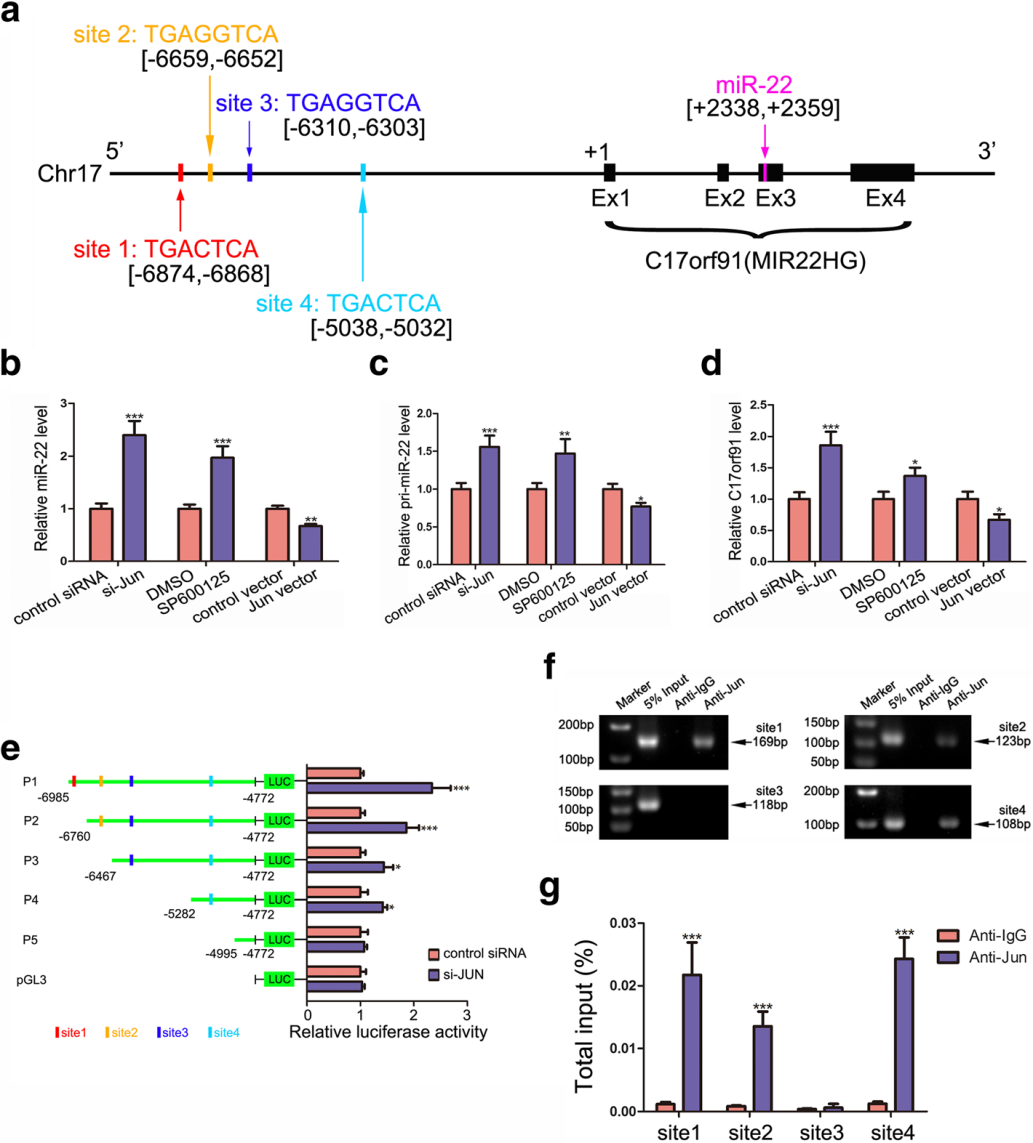
miR-22 is inhibited by Jun at the transcriptional level. **a** Schematic descriptions of the genomic location of miR-22 and Jun’s putative binding sites in the promoter region of miR-22 host gene C17orf91. **b**-**d** The influences of Jun on the levels of mature miR-22, pri-miR-22 and C17orf91, respectively. **e** Luciferase activities of different miR-22 promoter reporter constructs, co-transfected with si-Jun or a negative control. **f** and **g** ChIP assay for Jun’s occupancy on the C17orf91 promoter. *P < 0.05; **P < 0.01; ***P < 0.001

To uncover the mechanism for miR-22 downregulation in CRC, we first measured the levels of pri-miR-22 (the precursor of miR-22) in CRC tissues and found they were downregulated in CRC tissues compared with those in normal tissues (Additional file 7: Figure S5), indicating that miR-22 was transcriptionally silenced during CRC tumourigenesis. Transcription factors (TFs) are often dysregulated in cancers and closely associated with cancer progression [45, 46] and cancer-related miRNA expression [47]. Thus, we speculated that some TFs might regulate miR-22 transcription. Hsa-miR-22 is located in the 3rd exon of a non-coding transcript C17orf91 (also called MIR22HG) in chromosome 17, and it is co-transcribed with this host gene [48]. The potential promoter region (10 kb upstream the transcriptional start site) of C17orf91 was analysed using JASPAR [43] and SABiosciences [44]. Both software packages identified the onco-TF Jun as potential regulator of miR-22. According to the prediction, Jun could bind the promoter of C17orf91 at four possible sites (Fig. 6a). Next, we used siRNA or overexpression vectors to specifically knock down or raise Jun’s level (the efficiencies of si-Jun and Jun vectors are shown in Additional file 4: Figure S2b) and investigated whether Jun affected mature miR-22, pri-miR-22 and C17orf91 levels. As presented in Fig. 6b-d, inhibition of Jun increased the levels of mature miR-22, pri-miR-22 and C17orf91, and vice versa. Similarly, introduction of a JNK (c-Jun N-terminal kinase) specific inhibitor SP600125, which was used to inhibit Jun activity, significantly increased the expression levels of miR-22, pri-miR-22 and C17orf91 (Fig. 6b-d). These results indicated that Jun could suppress the transcription of miR-22.

To determine regulatory regions involved in miR-22 inhibition, several C17orf91 promoter regions containing different Jun binding sites were cloned into the pGL3 basic vector to perform promoter deletion analysis (Fig. 6e). With all four binding sites in vectors, silencing Jun enhanced luciferase activity, which remained stable when all the binding sites were lost. After deleting site 1, the increase in fluorescence signal was less than when site 1 was in the promoter, indicating the efficiency of site 1 in miR-22 regulation. A similar result was observed after deleting site 2 and site 4 gradually, but this did not occur with site 3, indicating that site 3 had little effect on the expression regulation of miR-22 (Fig. 6e). In addition, chromatin immunoprecipitation (ChIP) assays were performed to confirm the validity of the binding sites. As shown in Fig. 6f and g, Jun was successfully recruited by binding site 1, site 2 and site 4. However, Jun could not bind site 3. The results further confirmed that Jun transcriptionally repressed miR-22 by binding directly at site 1, site 2 and site 4 in C17orf91 promoter regions, but not site 3.

**Fig. 7:**
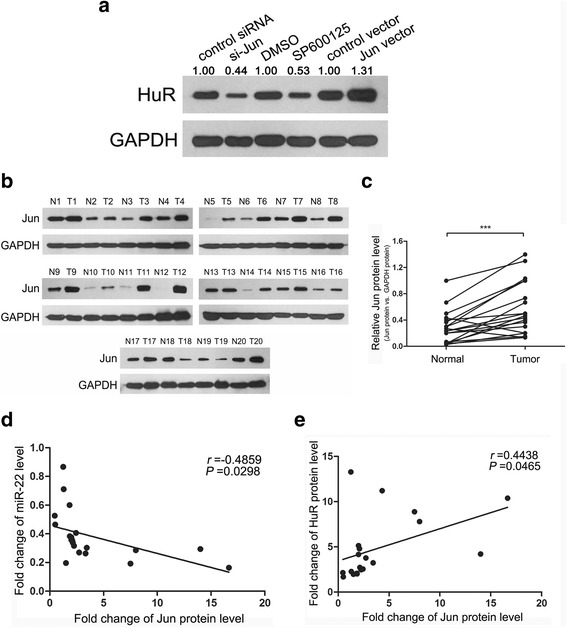
The Jun/miR-22/HuR axis exists in SW480 and CRC tissues. **a** Western blot analysis of HuR levels after altering Jun expression or activity in SW480. **b** and **c** Western blot analysis of HuR levels in CRC tissue pairs. **d** Pearson’s correlation scatter plot of the fold changes of Jun protein and miR-22 levels in CRC tissue pairs. **e** Pearson’s correlation scatter plot of the fold changes of Jun and HuR protein levels in CRC tissue pairs. ***P < 0.001

Finally, we tested if Jun could affect HuR expression by inhibiting miR-22. Inhibition of Jun expression or activity decreased HuR expression while overexpression of Jun increased HuR level (Fig. 7a), which is opposite from the alteration of miR-22 expression after the same treatment (Fig. 6b). We also analysed the relationship among Jun, miR-22 and HuR in CRC tissue samples. Jun protein levels increased in 18 of the 20 paired CRC tissues mentioned before (Fig. 7b and c). Pearson correlation analysis revealed a significant negative correlation between the expression of Jun and miR-22 (Fig. 7d), and a positive correlation between Jun and HuR (Fig. 7e), which supported the existence of Jun/miR-22/HuR axis in CRC.

Taken together, these results suggest that Jun negatively regulates the transcription of miR-22 via specific Jun-binding motifs in the promoter region of miR-22, through which Jun could enhance HuR expression.

## Discussion

HuR is a representative RNA binding protein that plays vital roles in CRC tumourigenesis [22–24]. In this study, significant upregulation of HuR was observed in CRC tissues compared with that in adjacent normal tissues, and high HuR levels predicted a lower survival rate among CRC patients. The oncogenic roles of HuR in CRC were also investigated, and the results indicated that HuR promoted CRC cell proliferation and migration in vitro and accelerated CRC tumour growth in vivo. Interestingly, the HuR mRNA and protein levels changed inconsistently in CRC samples, raising the possibility that HuR was regulated by aberrantly expressed miRNAs at the post-transcriptional level in CRC. Combining bioinformatics predictions and in vitro validation, miR-22 and miR-129 were demonstrated to be upstream repressors of HuR by directly binding to its 3’-UTR.

miRNAs are closely involved in CRC tumourigenesis. In every stage of CRC, there are many miRNAs that have been shown to have altered expression and are thus involved in the regulation CRC cancer hallmarks [49]. Among these myriad CRC-related miRNAs, miR-22 is one of the most important. miR-22 can affect various CRC phenotypes, including proliferation, migration, chemoresistance, apoptosis and angiogenesis [37–40]. Here, miR-22 was found to be markedly reduced in CRC, and lower miR-22 expression predicted a shorter life expectancy. miR-22 functioned as a tumour-suppressive miRNA in CRC to inhibit CRC proliferation and migration and tumour growth by targeting HuR. Our results highlighted the importance of miR-22 and HuR in CRC, and noted the possibility that targeting miR-22 or HuR might be a practical way to treat CRC in clinical environments. Kota et al. systemically delivered tumour-suppressive miR-26a in a mouse model by adeno-associated virus (AAV) to successfully treat hepatocellular carcinoma [50]. This strategy is also applicable to miR-22. For HuR intervention, besides administration of miR-22, a specific small molecule inhibitor or siRNA [51, 52] should be a practical and efficient treatment. More attention should be payed to the miR-22/HuR regulatory axis in CRC treatment.

Research on miRNAs has mainly focused on identifying the target genes of miRNAs. Indeed, these studies have been critical and necessary. However, as gene expression regulators, miRNAs themselves undergo complicated regulations in cancers [47, 53]. Cancer-associated transcription factors are key players in orchestrating gene expression networks in cancers, including miRNAs [45, 46]. Many TFs/miRNA regulatory pairs have been discovered, and their vital roles in cancer progression have been explored, such as P53/miR-34 and CMYC/miR-17-92 [54, 55]. Various downstream target genes of miR-22 have been elucidated, including HIF1α, VEGF, TIAM1, MMP-2, COX-2 [38–40], and HuR here. However, the causes for its downregulation in CRC are unknown. In this study, we demonstrated that miR-22 was directly repressed at the transcriptional level by the onco-TF Jun, which is a core member of transcription factor complex AP-1 involved in the oncogenesis of various cancers [56, 57]. The MAPK pathway (including MEK, ERK and p38) lies upstream of Jun, and can activate Jun expression [57]. One study reported that ERK can repress the expression of miR-22 [58]. Considering our results, this inhibition might be explained by ERK-activated Jun, which could then inhibit miR-22. Yang et al. reported that in ischaemia-reperfusion (I/R)-induced myocardial injury, miR-22 could repress the level of c-Jun-AP-1 and p-c-Jun-AP-1 by reducing p38 MAPK [59]. In another paper, miR-22 could significantly inhibit the DNA-binding ability of AP-1 [60]. These data suggested a double-negative regulatory relationship between miR-22 and Jun. It should be noted that Jun might also regulate miR-22 via an indirect mechanism. Some studies have reported that Jun can inhibit p53 expression and activity [61, 62], whereas p53 can transcriptionally activate miR-22 [63, 64]. There is the possibility that Jun/p53/miR-22 axis exists in CRC also.

AREs are widely distributed in the 3’-UTRs of protein-coding genes, including Jun [65]. Thus, Jun may be stabilised post-transcriptionally by HuR. This possibility was partially validated by a recent study, which revealed that HuR can increase Jun expression by binding one ARE in its 3’-UTR, and this effect can be reinforced by miR-200a [66]. This finding, combined with our results, suggests that Jun, miR-22 and HuR participate in a double-negative feedback loop in CRC cells. Because a double-negative feedback is equal to positive feedback and is known for its ability to amplify a response into a self-sustained mode that is independent of the original stimuli, the feedback loop composed of Jun, miR-22 and HuR may minimize miR-22 expression and amplify HuR expression in CRC cells, thus allowing CRC cells to become more autonomous, for example, to reproduce more rapidly and to metastasize to new microenvironments. Thus, this feedback regulation may explain the widespread downregulation of miR-22 and the overexpression of HuR in CRC.

## Conclusions

**Fig. 8:**
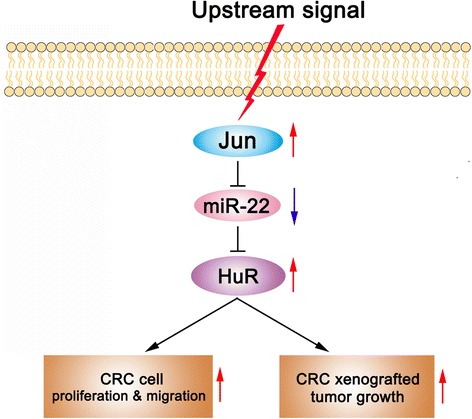
Working model of the Jun/miR-22/HuR axis in CRC

Taken together, this study identified an essential Jun/miR-22/HuR regulatory axis in CRC (the working model is summarised in Fig. 8) and highlighted the vital role of HuR and miR-22 in CRC proliferation and migration. The findings may provide attractive potential targets for CRC prevention and treatment.

## Additional files

Additional file 1: Table S1. Clinical features of colorectal cancer patients. (DOCX 17 kb)

Additional file 2: Table S2. Sequences of siRNAs, probe and primers. (DOCX 16 kb)

Additional file 3: Figure S1. HuR protein is significantly upregulated in CRC tissues and negatively correlated with CRC patient survival. (a) HuR levels in normal colon, normal rectum, colon adenocarcinoma and rectal adenocarcinoma in the TCGA dataset analysed by Oncomine. (b) Kaplan-Meier curve showing the negative correlation of HuR level and CRC patients’ survival. (TIFF 167 kb)

Additional file 4: Figure S2. The efficiencies of siRNA and overexpression vector of HuR (a) or Jun (b). (TIFF 377 kb)

Additional file 5: Figure S3. HuR functions as an oncogene in CRC. (a-c) HuR promoted SW480 proliferation. a: CCK-8 assays; b and c: EdU assays. (d and e) HuR promoted SW480 migration. (f-h) HuR accelerated CRC xenografted tumour growth. f: Photos of CRC tumours; g: Tumour volume curves; h: Tumour weights. (i) Western blot analysis of HuR levels in CRC xenografted tumours. (j and k) HE staining and IHC staining for HuR and Ki-67 in xenografted tumours. **P < 0.01; ***P < 0.001. (TIFF 5991 kb)

Additional file 6: Figure S4. (a and b) The Kaplan-Meier curve revealed the positive correlation of miR-22 (a) or miR-129 (b) level and CRC patients’ survival. (c and d) The transfection efficiencies of miR-22 (c) or miR-129 (d) mimics or inhibitors in 3 CRC cell lines. ***P < 0.001. (TIFF 1147 kb)

Additional file 7: Figure S5. Pri-miR-22 is downregulated in CRC tissues compared with that in normal adjacent tissues. *P < 0.05. (TIFF 186 kb)

## Abbreviations

3’-UTR: 3′-untranslated region
CRC: Colorectal cancer
H&E staining: Haematoxylin and eosin staining
HuR: Hu antigen R
IHC staining: Immunohistochemical staining
miRNA: microRNA
β-gal: β-galactosidase
