# Supplementary material for: The Jun/miR-22/HuR regulatory axis contributes to tumourigenesis in colorectal cancer
Source: Mol Cancer. 2018 Jan 19;17:11. doi: 10.1186/s12943-017-0751-3 (PMC5775639; doi:10.1186/s12943-017-0751-3)
Supplement: Supplementary file 1 — Clinical features of colorectal cancer patients. (DOCX 17 kb) [file 12943_2017_751_MOESM1_ESM.docx]

**The Jun/miR-22/HuR regulatory axis contributes to tumourigenesis in colorectal cancer**

**Additional file 1: Table S1. Clinical features of CRC patients**

| **Case number** | **Age** | **Gender** | **TNM stage** | **Cancer subtype** |
| --- | --- | --- | --- | --- |
| 1 | 56 | M | III | rectal carcinoma |
| 2 | 43 | M | IV | colon carcinoma |
| 3 | 39 | F | III | colon carcinoma |
| 4 | 66 | F | III | rectal carcinoma |
| 5 | 47 | F | II | colon carcinoma |
| 6 | 56 | M | II | colon carcinoma |
| 7 | 40 | M | IV | rectal carcinoma |
| 8 | 62 | F | II | colon carcinoma |
| 9 | 66 | F | III | colon carcinoma |
| 10 | 65 | M | III | colon carcinoma |
| 11 | 71 | F | IV | colon carcinoma |
| 12 | 47 | M | II | colon carcinoma |
| 13 | 59 | M | III | colon carcinoma |
| 14 | 64 | F | III | colon carcinoma |
| 15 | 61 | M | II | colon carcinoma |
| 16 | 59 | F | II | rectal carcinoma |
| 17 | 45 | F | III | colon carcinoma |
| 18 | 67 | M | III | rectal carcinoma |
| 19 | 70 | M | II | colon carcinoma |
| 20 | 61 | F | III | colon carcinoma |
