# Supplementary material for: The Jun/miR-22/HuR regulatory axis contributes to tumourigenesis in colorectal cancer
Source: Mol Cancer. 2018 Jan 19;17:11. doi: 10.1186/s12943-017-0751-3 (PMC5775639; doi:10.1186/s12943-017-0751-3)
Supplement: Supplementary file 2 — Sequences of siRNAs, probe and primers. (DOCX 16 kb) [file 12943_2017_751_MOESM2_ESM.docx]

**The Jun/miR-22/HuR regulatory axis contributes to tumourigenesis in colorectal cancer**

**Additional file 2: Table S2. Sequences of siRNAs, probe and primers**

| **Item** | **Sequence** |
| --- | --- |
| si-HuR | AAGAGGCAATTACCAGTTTCA |
| si-Jun | AGTCATGAACCACGTTAAC |
| HuR pull down probe | GCAAGGATCGCGCACACAGCCCCTCAGT |
| HuR qRT-PCR primers | Forward：GGGTGACATCGGGAGAACGAAT |
|  | Reverse：TGTCCTGCTACTTTATCCCGAA |
| pri-miR-22 qRT-PCR primers | Forward：AGAGCCTTTGCCCAGATT |
|  | Reverse：GGGTTGTTTGAGCCTTCTAC |
| C17orf91 qRT-PCR primers | Forward：TTCCTGGATGACAGTTGG |
|  | Reverse：AAACCATTGCTTCCCATT |
| Jun binding site 1 ChIP primers | Forward：GGCACAGGATATGGAATAG |
|  | Reverse：TCCTTCAAGAGGCAAACT |
| Jun binding site 2 ChIP primers | Forward：TGGCCGGTCACGGTGGTTCA |
|  | Reverse：TTTTAGTAGAGATGGAGTTTC |
| Jun binding site 3 ChIP primers | Forward：TGCGGTGGCTCATGCCTGTA |
|  | Reverse：TATTTTAGTAGAGATGGGGT |
| Jun binding site 4 ChIP primers | Forward：AAAGTTTCGCTCTTGTTGCC |
|  | Reverse：TACTCGGGATGCTGAGGC |
