# Supplementary figures and images for: The Jun/miR-22/HuR regulatory axis contributes to tumourigenesis in colorectal cancer
Source: Mol Cancer. 2018 Jan 19;17:11. doi: 10.1186/s12943-017-0751-3 (PMC5775639; doi:10.1186/s12943-017-0751-3)

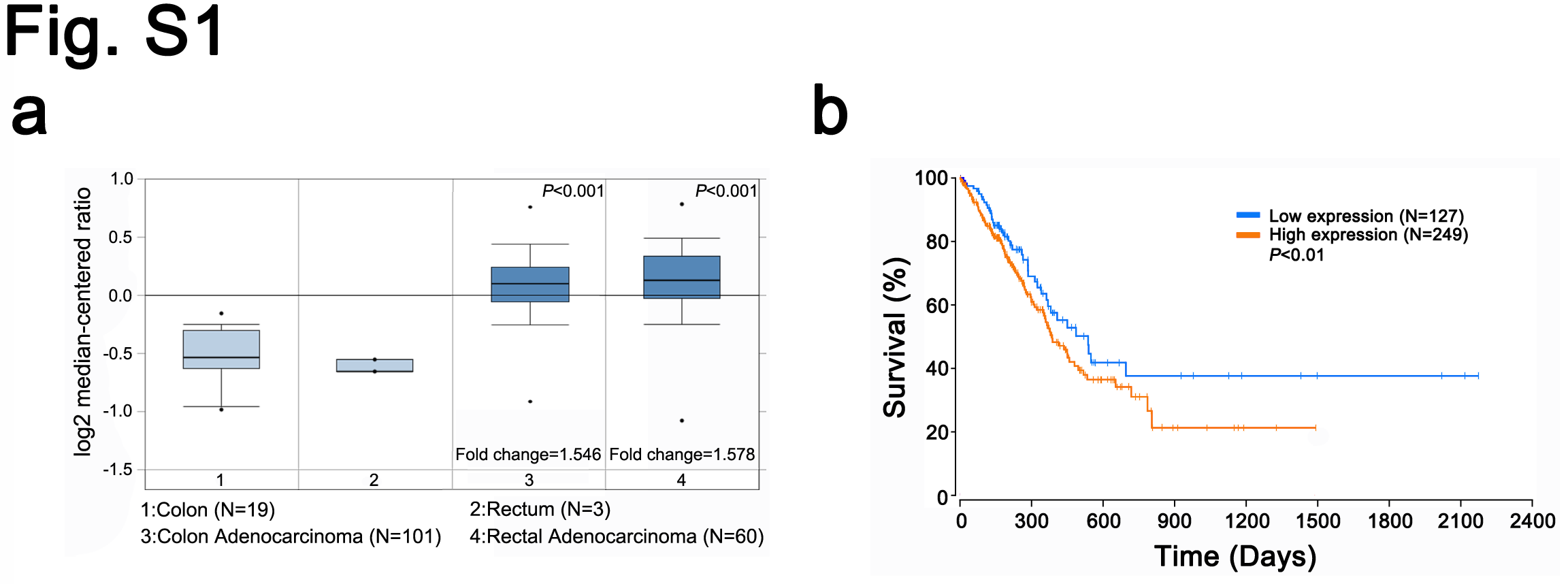

Supplement: Supplementary file 3 — HuR protein is significantly upregulated in CRC tissues and negatively correlated with CRC patient survival. (a) HuR levels in normal colon, normal rectum, colon adenocarcinoma and rectal adenocarcinoma in the TCGA dataset analysed by Oncomine. (b) Kaplan-Meier curve showing the negative correlation of HuR level and CRC patients’ survival. (TIFF 167 kb) [file 12943_2017_751_MOESM3_ESM.tif]

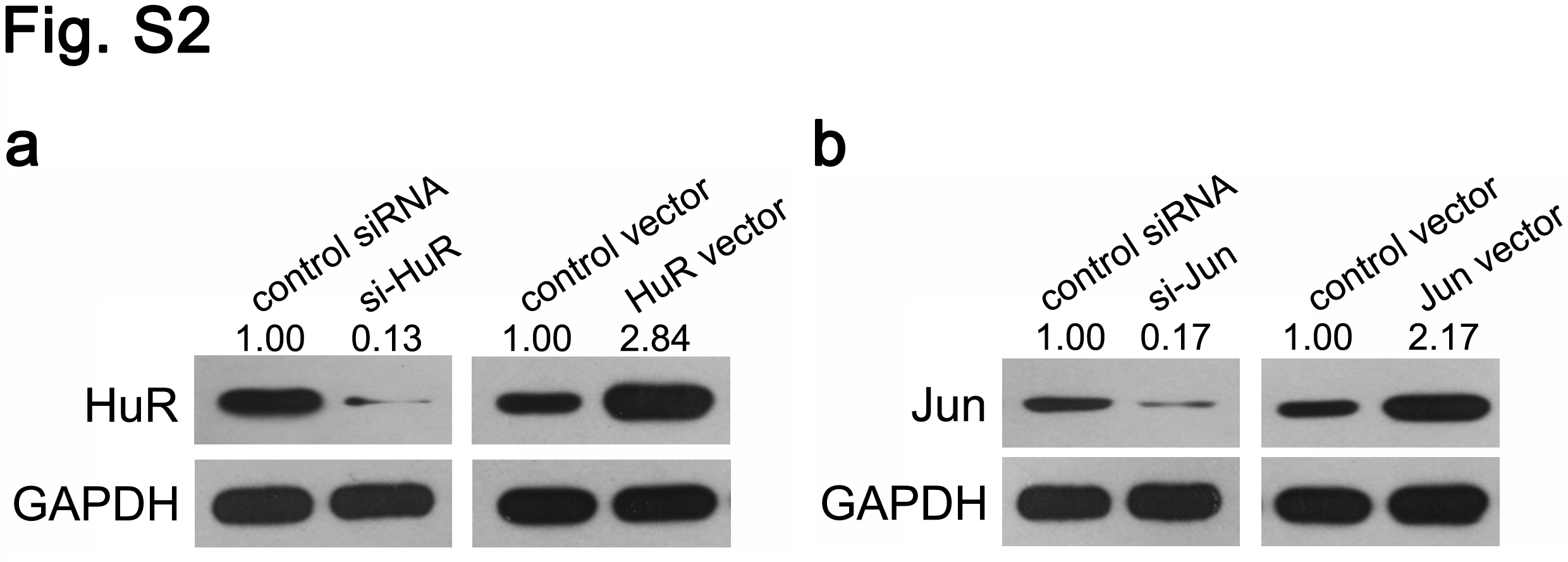

Supplement: Supplementary file 4 — The efficiencies of siRNA and overexpression vector of HuR (a) or Jun (b). (TIFF 377 kb) [file 12943_2017_751_MOESM4_ESM.tif]

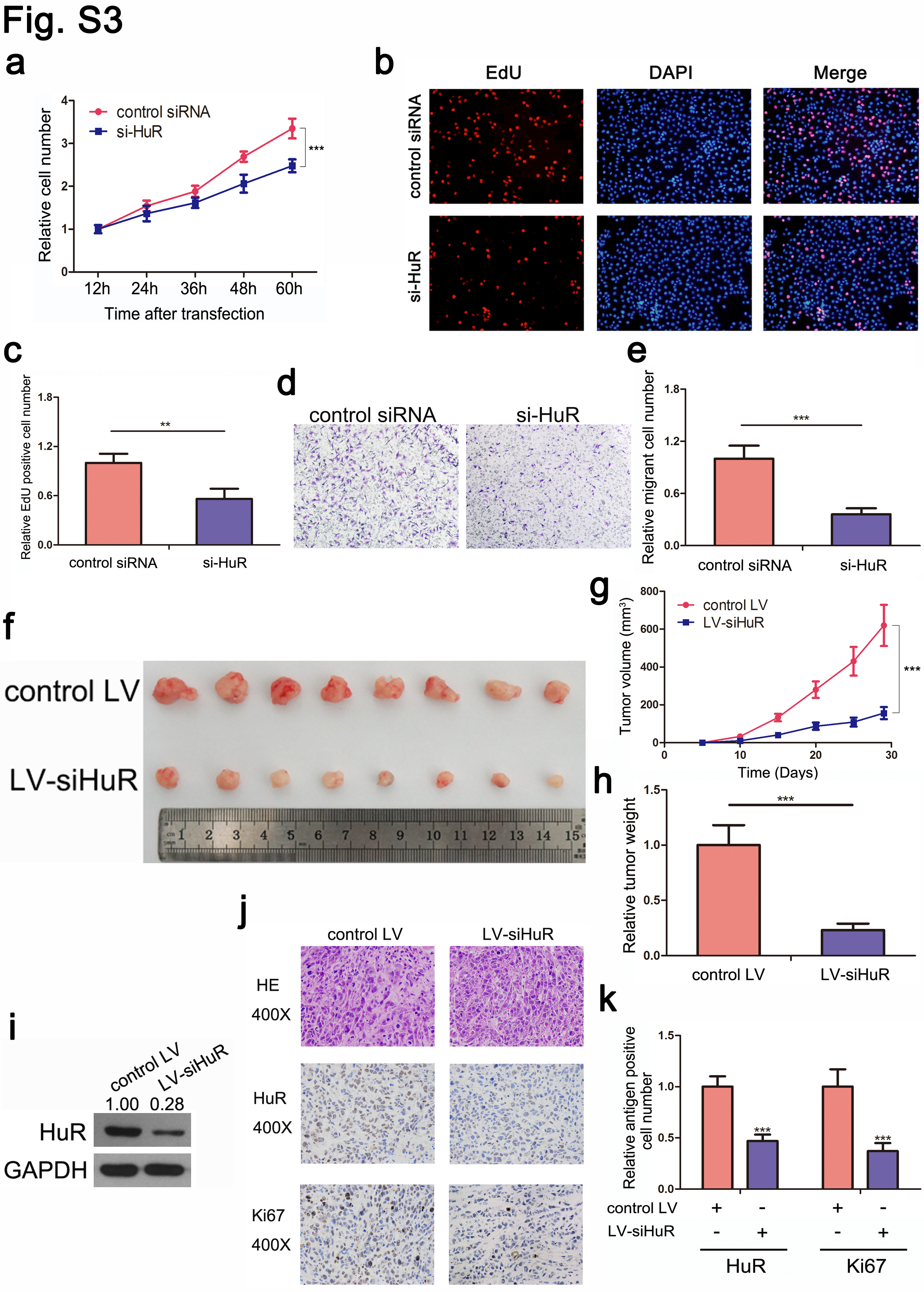

Supplement: Supplementary file 5 — HuR functions as an oncogene in CRC. (a-c) HuR promoted SW480 proliferation. a: CCK-8 assays; b and c: EdU assays. (d and e) HuR promoted SW480 migration. (f-h) HuR accelerated CRC xenografted tumour growth. f: Photos of CRC tumours; g: Tumour volume curves; h: Tumour weights. (i) Western blot analysis of HuR levels in CRC xenografted tumours. (j and k) HE staining and IHC staining for HuR and Ki-67 in xenografted tumours. **P < 0.01; ***P < 0.001. (TIFF 5991 kb) [file 12943_2017_751_MOESM5_ESM.tif]

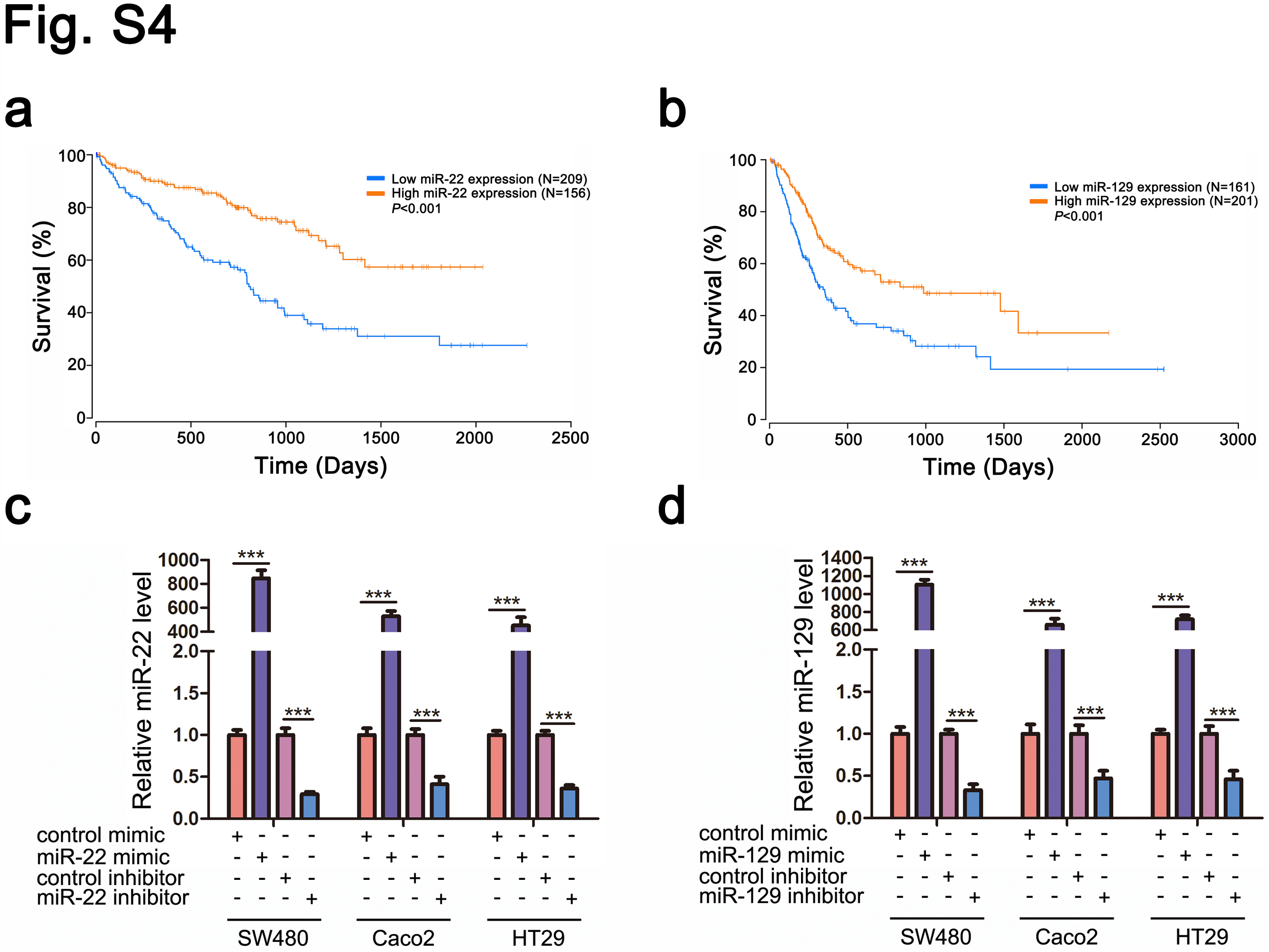

Supplement: Supplementary file 6 — (a and b) The Kaplan-Meier curve revealed the positive correlation of miR-22 (a) or miR-129 (b) level and CRC patients’ survival. (c and d) The transfection efficiencies of miR-22 (c) or miR-129 (d) mimics or inhibitors in 3 CRC cell lines. ***P < 0.001. (TIFF 1147 kb) [file 12943_2017_751_MOESM6_ESM.tif]

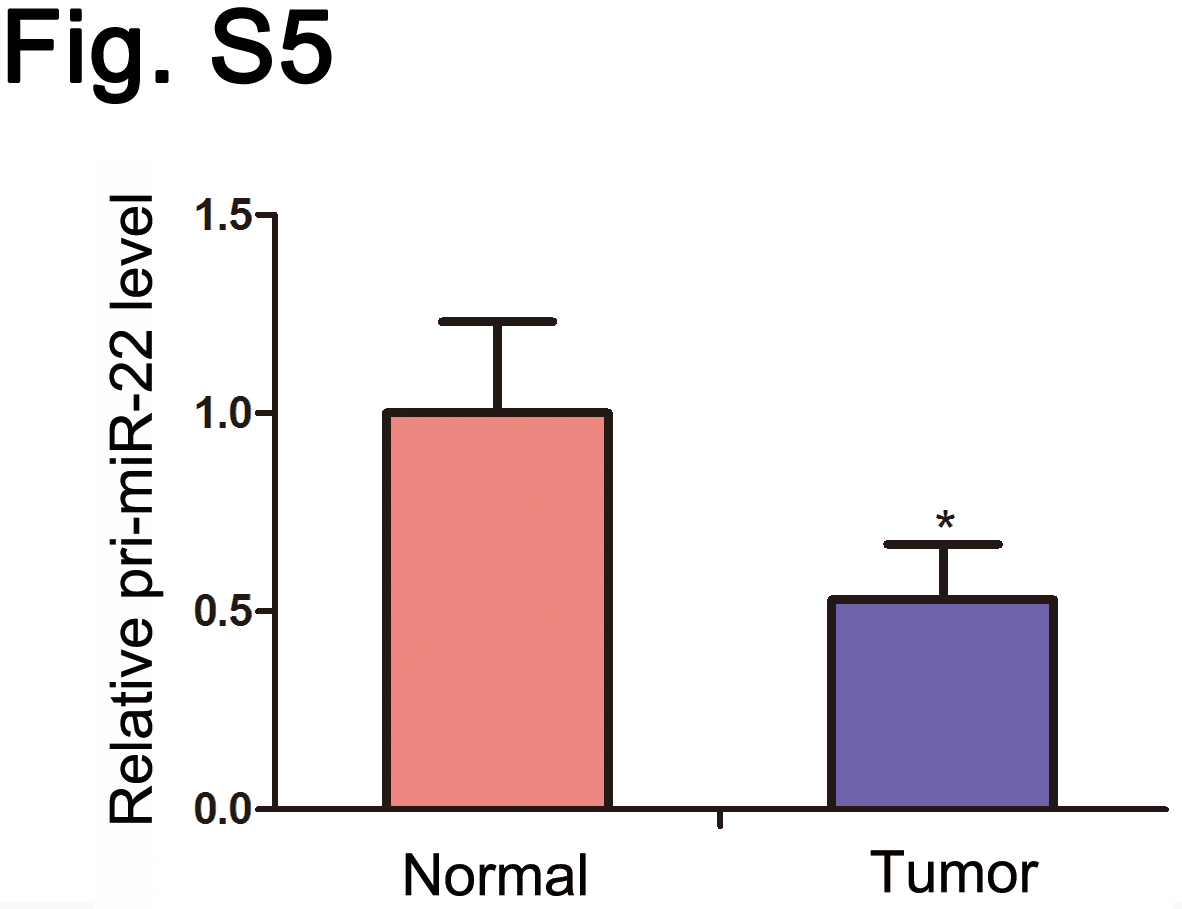

Supplement: Supplementary file 7 — Pri-miR-22 is downregulated in CRC tissues compared with that in normal adjacent tissues. *P < 0.05. (TIFF 186 kb) [file 12943_2017_751_MOESM7_ESM.tif]
